# Pain Management in a Terminally Ill Patient with a Surrogate Decision-maker: A Challenge

**DOI:** 10.7759/cureus.5429

**Published:** 2019-08-19

**Authors:** Tausif Syed, Susan Mansourian, Pratyusha Tirumanisetty, Abdullah Abdullah, Richard Alweis

**Affiliations:** 1 Internal Medicine, Rochester Regional Health / Unity Hospital, Rochester, USA; 2 Internal Medicine, Rochester Regional Health, Rochester, USA; 3 Internal Medicine, Rochester Region Health, Rochester, USA; 4 Internal Medicine, University of Rochester School of Medicine and Dentistry/ Unity Health System, Rochester, USA

**Keywords:** pain management, surrogate decision maker, opioids

## Abstract

When health care proxies are in charge of pain management, it may become very difficult to address the patient’s pain if the health care proxy has misconceptions about analgesics.

We report a case of an 87-year-old lady who was found to be pulseless, and after successful cardiopulmonary resuscitation (CPR), was intubated and remained so for over a month in the intensive care unit (ICU). The medical team could not provide the patient with pain medication, as the daughter who was the surrogate decision-maker did not allow the administration of any pain medication in the false belief that it would kill the patient.

This case helps us to shed light on the surrogate decision-maker and pain management and how medical professionals can solve similar issues in the future. Early consultation with palliative care may be beneficial. For continued disagreement, the ethics committee should be consulted.

## Introduction

Pain management remains one of the most important, yet controversial, factors in high-quality, evidence-based patient care. Pain management is a multifactorial process with contributions from patients, families, and healthcare providers while the role of regulators and hospital policy must also be considered [1]. This is especially important in the case of patients who are not capable of communicating their true level of pain to the provider. When health care proxies are in charge of pain management, it may become very difficult to address the patient’s pain if the health care proxy has misconceptions about analgesics [2]. Medical providers must seek to understand the reasoning behind the health care proxy refusing pain medication and address any concerns and misconceptions.

## Case presentation

An 87-year-old female, with a past medical history significant for anemia, atrial fibrillation (not on anticoagulation), mitral and aortic regurgitation, ischemic cardiomyopathy (left ventricular ejection fraction 15%), hyperlipidemia, and hypertension, was found to be lethargic and confused by a visiting family member. Emergency medical services (EMS) was activated and upon their arrival, the patient was found to be pulseless. Cardiopulmonary resuscitation (CPR) was initiated and after two minutes, a return of spontaneous circulation (ROSC) was achieved. She was intubated, admitted to the intensive care unit (ICU), and managed for aspiration pneumonia and sepsis. Upon stabilization after a few days, she was extubated and transferred to the floor. During the recovery period on the medical floor, her condition deteriorated once again. She was found to have intestinal perforation and pneumoperitoneum.

Surgical intervention was not appropriate due to significant comorbidities. She was started on conservative management but suffered respiratory and renal failure. She had to be re-intubated urgently and transferred back to the ICU. In the ICU, the patient remained intubated. Despite all efforts, her pulmonary function did not improve, and it was not possible to extubate her. It was obvious from her facial expressions that she was in pain and distress. Her daughter, who was the healthcare proxy, elected to continue full code status and would not allow any analgesics, opioid or otherwise, to be used for comfort. She was under the impression that analgesics, especially of the opioid family, would hasten her mother’s death. She also emphasized that the patient was intubated without any analgesics and, therefore, did not require any pain medication after intubation. Multiple meetings between the daughter, other members of the family, the ICU team, and the palliative team failed to convince the daughter that while intubated, the patient could be in true pain and require pain management. In various meetings, it came to light that the daughter had lost her father one month back and was not ready to lose her mother at this time. This fear that she might lose her mother and that pain medications could increase the chance of death, however small, led her to deny the use of any pain medications. The other family members, including the patient’s son, came to the meeting. They were also of the opinion that the patient should receive the pain medication but since the daughter was the sole health care proxy, the final decision was with the daughter. The patient was also offered other forms of analgesia, including an epidural and regional block, but those were also denied by the daughter. The ethics committee consultation indicated there was no route to bypass the health care proxy’s wishes, even if the medical team felt it was for the patient’s benefit. The patient remained full code, intubated, partially awake, and in what the medical team felt was obvious pain for over a month in the ICU before passing away.

## Discussion

Pain relief is the legal right of patients as declared in a number of cases by the Supreme Court of USA, and medical providers are ethically responsible to assure adequate pain management. The international community has declared pain relief as a basic human right [1]. Almost 35%-75% of seriously ill patients experience severe pain [2]. It is not only the healthcare proxies but also, occasionally, physicians who have a tendency to fear using opioids [3-4].

The main reason for this fear in terminally ill patients is the belief that increasing the doses of opioids may accelerate death and/or become a legal liability for the provider [5]. Both of these beliefs are wrong. According to a study, there is no difference between the increasing doses of opioids and stable doses of opioids in the survival of a terminally ill patient [6-8]. The second fear, which is more specific to providers, is the possibility of getting sued for causing addiction or hastening death by using opioids. This is also a misconception, as the Supreme Court of the USA has asked all the states to avoid any law that would be a hindrance to adequate palliative care [9]. The New York State Task Force on Life says that it is acceptable to use analgesics even if they hasten death if the pain medications were prescribed to decrease the pain and not with the intention of causing death [10].

Patients and their healthcare proxies have the right to refuse any treatment [6,11]. The decisions taken by the healthcare proxy are accepted, as they are believed to be in alignment with the patient’s wishes. Despite having control over the decision-making, the healthcare proxy’s decisions should be reasonable. It is indisputable that analgesics decrease the burden of disease on the patient. A reasonable decision would be to allow the use of pain medications, especially while the patient is intubated. In this instance, refusal of the pain medication by the health care proxy was not in the best interests of the patient. As Massachusetts legislators have said in the Health Care Proxy Law, the surrogate does not have the power to deny the use of pain medications, sedatives, non-oral hygienic care, etc. that the medical team deems necessary [12]. In our patient, the New York state where this case was located does not have such law, which could have allowed the medical team to provide the pain medication and bypass the surrogate decision-makers wishes.

The solution to this impasse is continuous negotiation [13]. This helps with the patient maintaining autonomy as well as the medical team providing the relief that the patient requires. This negotiation should begin at the earliest time possible, as it will help to develop the bond between the patient and the medical team [14]. Studies have shown that making decisions regarding a patient’s management is very stressful for the healthcare proxy [15]. During these stressful times, the decision-making process is compromised. In our case, there were a number of discussions with the patient’s daughter but no compromise could be reached. One part of the negotiation is the education of the patient’s family regarding the benefits of adequate pain control and the removal of the fear of using opioids or other forms of pain relief. The patient's family should be advised that increasing the dose of opioids do not necessarily hasten death [16]. Any misgivings regarding the use of opioids, such as misinformation presented by the mainstream media, should be sought early. The other reason for not using opioids may be cultural. Various studies have shown that non-Caucasians are less likely to trust the medical team regarding the management of the patient [17]. Asians and Hispanics are more likely to make a combined family decision rather than an individual decision when important medical decisions need to be made [17]. These points should be kept in mind before the medical team approaches the family regarding end-of-life decision-making and the use of pain medications. Early palliative care team involvement might have helped in this case as shown by previous studies. When these measures fail, the healthcare proxy can be a barrier to effective pain control.

The last resort is to request an ethics consultation. The ethical committee can guide to resolve the issue. However, for these consultations to be effective, clear guidelines need to be set. Some hospitals have recommended a clear framework for the ethical committee, which can help resolve these types of conflicts. These reports suggest that the ethical committee should assess whether the treatment is beneficial to the patient. If it is seen that the treatment is clearly beneficial, then a recommendation is made to start the treatment. If the decision is against the wishes of the health care proxy, they are given the option of transferring the patient to another facility. If the decision is against a certain provider’s recommendation, the patient may be transferred to the care of another provider in the same facility [18]. Unfortunately, no such clear guidelines were set before this case. As a result, the ethical committee could not come to a conclusion that would be acceptable to both the parties.

## Conclusions

Pain management under the care of health care proxy remains an area of clinical debate and ethical controversy. It is the duty of clinicians to provide the health care proxy with adequate background information to allow them to make a decision in alignment with the patient’s wishes. Early consultation with palliative care may be beneficial. For continued disagreement, the ethics committee should be consulted.
